# Characterization of Functional Properties and Organic Acids in *Vangueria infausta* Burch. (Wild Medlar/Nzvirumombe/Umviyo) Fruit(s) Found in Zimbabwe

**DOI:** 10.1155/ijfo/9925294

**Published:** 2025-04-08

**Authors:** P. Nemapare, D. T. Mugadza, T. H. Gadaga

**Affiliations:** ^1^Department of Food Science and Nutrition, Midlands State University, Gweru, Zimbabwe; ^2^Department of Environmental Health Science, University of Eswatini, Mbabane, Eswatini

**Keywords:** functional properties, health benefits, indigenous fruits, organic acid, phytochemicals, velvet wild medlar, volatile compounds

## Abstract

Edible indigenous wild fruits are popular in the communities they are found. In recent years, these fruits have been purveyed in urban markets and contribute to household income. However, the nutritional and other health benefits of some of the fruits have not been studied. The objectives of the current study were to detect and identify the bioactive compounds that give *Vangueria infausta* fruits their unique flavor characteristics and proposed health benefits. The study detected and quantified the phytochemical compounds present and profiled the volatile organic compounds and organic acids in the fruit. The phytochemicals detected were phenols, tannins, flavonoids, anthraquinones, alkaloids, coumarins, terpenoids, glycosides, anthocyanins, and quinones, which were extracted in both methanolic and aqueous solutions. Antioxidant activity was determined using the DPPH scavenging assay, and volatile compounds were determined using gas chromatography. Organic acids were determined using HPLC. It was observed that the *V. infausta* fruits contained phenols, tannins, flavonoids, coumarins, alkaloids, anthocyanins, and terpenoids. The concentration of total phenolic compounds, total flavonoids, and total tannins in methanolic extracts was significantly (*p* ≤ 0.05) higher than those in the aqueous extracts. The total phenolic content (TPC) of *V. infausta* fruit pulp was found to be in the range 128–170 mg GAE 100 g^−1^ dry weight basis, which corresponded to the low antioxidant activity of less than 20% that was recorded. Formic acid (68.25 mg/kg) and ascorbic acid (297.3 mg/kg) were the two organic acids detected in the fruit pulp. Various volatile compounds were also detected in the fruit pulp including 5-hydroxymethylfurfurals and fatty acids such as ethyl octadecanoic acid, methyl stearate, methyl and ethyl hexadecanoic acid, and methyl-2-furoate. Methyl stearate, hydroxymethyl furfurals, and methy-2-furoate were novel compounds in the current study. It was therefore concluded that the *V. infausta* fruits contained bioactive compounds that are important for its flavor and have both pharmacological and food processing applications. Further studies are needed to investigate options for value addition, propagation, and conservation of the fruit *V. infausta*.

## 1. Introduction

*Vangueria infausta* Burch. subsp. *infausta* is an indigenous fruit that is commonly consumed in Zimbabwe and the sub-Saharan Africa. It belongs to the family Rubiaceae [1]. Like many indigenous fruits, *V. infausta* fruits are underutilized, and a significant portion is lost during harvesting. It is widely distributed in Zimbabwe [2] and flowers from September to November. The fruits ripen from November to April [3]. The fruit has a pleasant, sweet–sour, mealy taste which is similar to that of apples. Fresh or dried fruits are traditionally consumed raw or processed into different products such as jams and juice or as a flavoring agent in porridge [4]. *Vangueria infausta* Burch. is also used for medicinal and nutritional purposes [1]. Maroyi [1] indicated that apart from the root, seed, stem, and bark that are used as medicine to treat various illnesses, *V. infausta* fruits can also be used to treat menstrual pain and parasitic worms. Gwatidzo et al. [3] also reported that *V. infausta* fruits have antimicrobial and anti-inflammatory activity. Phytochemical and volatile compound characterization of *V. infausta* fruits was reported in studies done in Mozambique, Botswana, Malawi, South Africa, and Zambia [5–8]. However, similar studies have not been done with Zimbabwean fruits. The nutritional, mineral, and chemical profiles of fruits from the same tree species can vary according to geographic location [9, 10]. The current study aimed to determine phytochemical and volatile organic compounds (VOCs) and organic acid content in Zimbabwean *V. infausta* fruits and assess the potential of this wild delicacy for use as a nutraceutical and flavoring agent in foods.

## 2. Materials and Methods

### 2.1. Collection of Fresh Fruits and Sample Preparation

Ripe *V. infausta* fruits (40 kg) were purchased from local vendors at Ascot market in Gweru City, Zimbabwe, in April 2021. The fruits were taken to the Department of Food Science and Nutrition at Midlands State University for analysis. Undamaged and untainted fruits were selected and thoroughly washed to remove dirt. The fruits were stored in a freezer (−18°C) before further processing and analysis. During preparation for analysis, the frozen fruits (ca. 2 kg) were defrosted for 6 h. For some tests, the fruit pulp was dried and ground into powder. The samples were analyzed in duplicates and triplicates depending on the test and availability of chemical standards.

### 2.2. Phytochemical Screening

The method described by Sharma et al. [11], with modifications, was adopted. To prepare the extracts, each dried fruit powder sample (50 g) was weighed and soaked in 50 mL of distilled water and methanol (1:1 *v*/*v*) in conical flasks. The suspension was shaken occasionally using a shaking incubator and macerated for 48 h at room temperature. The solution was percolated through cotton wool, and the filtrate/fruit extract was used for analysis.

#### 2.2.1. Test for Phenols

To the diluted extract, 3–4 drops of 10% ferric chloride were added. A dark-green color signified the presence of phenolic compounds.

#### 2.2.2. Test for Tannins

Braymer's test was used according to Vizhi et al. [12]. About 3–4 drops of 10% ferric chloride were added. A blue color indicated the presence of gallic tannins, and a green color showed presence of catechol tannins.

#### 2.2.3. Test for Flavonoids

The method by Shaikh and Patil [13] was used. The filtrate was added to 5 mL dilute ammonia solution. A few drops of concentrated sulphuric acid were then added. A yellow color showed presence of flavonoids.

#### 2.2.4. Test for Anthraquinones

Borntrager's test method by Khan et al. [14] was used. A few drops of the extract filtrate were mixed with 10 mL of 10% ammonia solution. The mixture was shaken vigorously for 30 s. A pink, violet, or red color signified presence of anthraquinones.

#### 2.2.5. Test for Coumarins

The sodium hydroxide test was used [12]. The fruit extract was mixed with 10% sodium hydroxide and chloroform. A yellow color signified the presence of coumarins.

#### 2.2.6. Test for Alkaloids

Meyer's test was used according to Talukdar and Chaudhary [15] method. To a portion of the extract (2 mL), 1 mL of Meyer's reagent (solution A: 1.358 g mercuric chloride+60 mL distilled water mixed with solution B: 5 g potassium iodide+10 mL distilled water and made up to 100 mL with distilled water) was added. The presence of a pale yellow precipitate indicated the presence of alkaloids.

#### 2.2.7. Test for Terpenoids

The method described by Alamzeb et al. [16] was used. The fruit extract (5 mL) was mixed with 2 mL chloroform and 3 mL concentrated sulphuric acid. Reddish brown coloration indicated the presence of terpenoids.

#### 2.2.8. Test for Glycosides

This was done using the method described by Shaikh and Patil [13]. The fruit extract/filtrate (5 mL) was mixed with glacial acetic acid (2 mL). A drop of 5% FeCl_3_ and concentrated sulphuric acid were then added. A brown ring indicated the presence of glycosides.

#### 2.2.9. Test for Anthocyanins

The HCl test method reported by Shaikh and Patil [13] was used. The fruit filtrate (2 mL) was mixed with 2 N HCl (2 mL) and a few drops of ammonia. A pink–red color which turns blue–violet after addition of ammonia indicated the presence of anthocyanins.

#### 2.2.10. Test for Quinones

The sulphuric acid test by Iheagwam et al. [17] was used. One drop of concentrated sulphuric acid was added to 10 mg of each dried fruit powder dissolved in isopropyl alcohol. Formation of red color indicated the presence of quinones.

### 2.3. Quantitative Determination of Phytochemicals

#### 2.3.1. Total Phenolic Compounds

A method reported by Bhebhe et al. [18], with modifications, was used. Two different solvents were used for extraction, distilled water, and methanol. Dried fruit powder (1 g) was mixed with 9 mL of each of distilled water and methanol separately. The mixture was vortexed for 1 min and centrifuged at 4000 rpm for 10 min using a Biobase BKC-MH12-B centrifuge (Biobase Meihua, Jinan, Shangdong, China). The supernatant was used for subsequent analysis. Each sample was analyzed in triplicate. Distilled water (950 *μ*L) was added into a 10-mL test tube containing each sample (50 *μ*L) to make up to 1 mL using a micropippette. Sodium carbonate (2%) (2.5 mL) was added followed by Folin–Ciocalteu reagent (500 *μ*L) (0.62 g/mL). The mixture was stored in a dark cupboard for 40 min. Absorbance was then measured at 725 nm on a 756S UV-Vis Spectrophotometer (Hinotek, Ningbo, China). Different concentrations of gallic acid (0.5 mg/mL) were used for preparation of a standard curve. Total phenolic content (TPC) was expressed as milligram of gallic acid equivalents per 100 g dry weight (mg GAE/100 g DW).

#### 2.3.2. Flavonoids

The method as described by Cosmulescu et al. [19] with modifications was used. Total flavonoids were determined using the aluminium nitrate colorimetric method. Dried fruit powder (1 g) was mixed with 9 mL of each of distilled water and methanol separately. The mixture was vortexed for 1 min and centrifuged at 4000 rpm for 10 min using a Biobase BKC-MH12-B centrifuge (Biobase Meihua, Jinan, Shangdong, China). Extracted supernatant was used for subsequent analysis. Each sample was analyzed in triplicate. The fruit extract (0.5 mL) was mixed with 10% aluminium nitrate (0.1 mL), 1 M aqueous potassium acetate (0.1 mL), and methanol (4.3 mL). After keeping it for 40 min at room temperature, the absorbance of the reaction mixture was measured at 415 nm. Quercetin was used to prepare a standard curve (0–100 mg/L). The results were expressed as milligrams of quercetin equivalents per 100 g dry weight (mg QE/100 g DW).

#### 2.3.3. Tannins

The method described by Bhebhe et al. [18], with modifications, was used. Dried fruit powder (1 g) was mixed with 9 mL of each of distilled water and methanol separately. The mixture was vortexed for 1 min and centrifuged at 4000 rpm for 10 min using a Biobase BKC-MH12-B centrifuge (Biobase Meihua, Jinan, Shangdong, China). Extracted supernatant was used for subsequent analysis. Each sample was analyzed in triplicate. Fruit extract (1 mL) was added to 16-mm test tubes. Butanol: HCl 95/5 (*v*/*v*) reagent mixture (3 mL) and ferric reagent (2% of ferric ammonium sulphate dissolved in 2 N HCl (0.1 mL)) were then added. The tubes were vortexed, covered with a glass marble, and heated in a water bath at (90°C–100°C) for 1 h. Test tubes were cooled, and absorbance was read at 550 nm using a 756S UV-Vis Spectrophotometer (Hinotek, Ningbo, China). Tannin content was calculated as leucocyanidin equivalent (LE) according to the formula:

 $$\begin{array}{c} C=\left[A\;\left(550\text{ nm}\right)\right]\times \text{dilution factor }\varepsilon ƅ, \end{array}$$

where *A* (550 nm) is the absorbance at 550 nm, *C* is the concentration in (LE/100 g), *ε* is 460 (extinction coefficient), and *ƅ* is 1 cm (path length of light).

### 2.4. Biochemical Tests

#### 2.4.1. Antioxidant Activity DPPH Scavenging Assay

The method by Bhebhe et al. [18] with modifications was used. Dried fruit powder (1 g) was mixed with 9 mL of each of distilled water and methanol separately. The mixture was vortexed for 1 min and centrifuged at 4000 rpm for 10 min using a Biobase BKC-MH12-B centrifuge (Biobase Meihua, Jinan, Shangdong, China). The supernatant was used for subsequent analysis. Each sample was analyzed in triplicate. Methanolic solution of 0.003% (*v*/*v*) DPPH (2950 *μ*L) was added to a cuvette. About 50 *μ*L of each diluted sample extract was mixed with the DPPH solution to make up to 3 mL. Absorbance value was measured at 517 nm using a 756S UV-Vis Spectrophotometer (Hinotek, Ningbo, China). Change in absorbance was measured for 10 min. Boiled distilled water was used as the negative control and gallic acid (0–1000 *μ*g/mL) as the positive control. Percentage radical scavenging activity was calculated using the formula:

 $$\begin{array}{c} \text{RSA}\left(\%\right)=\left\{1-\left[\frac{A_{\text{extract}}}{A_{\text{negative⁣control}}}\right]\right\}\times 100, \end{array}$$

where *A*_negative⁣control_ is the absorbance of the negative control and *A*_extract_ is the absorbance of extract at 517 nm after 10 min.

#### 2.4.2. Characterization of Phytochemicals Using the High Performance Liquid Chromatography

The phytochemical profiles were determined using an Agilent 1260 Infinity HPLC with an Eclipse PLUS C18 column (4.6 × 150 mm (internal diameter × length) and 3.5 *μ*m diameter of particles). Fruit powder (10 g) was refluxed with methanol/water (50:50 *v*/*v*) for 16 h using a Soxhlet extractor. The extract was then evaporated to dryness under vacuum in a rotary evaporator. The dried extract was dissolved in 10 mL methanol, filtered, and injected into the HPLC. Water adjusted to pH 2.5 with orthophosphoric acid (98:2 *v*/*v*) was used as Solvent A, while acetonitrile was Solvent B. Both solvents were mixed (A 60:B 40) with an isocratic elution of 60% A+40% B for gallic acid. Methanol was used as Solvent B for caffeic acid, vanillin, coumaric acid, and quercetin and mixed with Solvent A. Gradient elution was used to separate the components in the following way: 10% B at 0 min, 10% B at 2 min, 90% B at 22 min, 90% B at 27 min, and 10% B at 28 min. Injection volume was 1.00–5 *μ*L as shown in Table 1. The flow rate was 0.5 mL/min at 35°C. Spectral data for all peaks ranged from 100–560 nm measured using Diode Alloy Detector. Absorbance was determined at 270, 300, and 285 nm. Pure standards purchased from Merck, South Africa, for gallic acid, coumaric acid, caffeic acid, quercetin, and vanillin were used for calibration. The tests were done in duplicates.

#### 2.4.3. Determination of Volatile Compounds Using Gas Chromatography (GC)

VOCs were determined using a 7890A GC system (Agilent Technologies) equipped with a split/splitless (1:100) capillary injector and a 5975C VL MSD with Triple-Axis detector (Agilent Technologies). Analytical separation was carried out on an HP-5MS capillary column (30 m × 0.25 mm i.d.) (Agilent Technologies). The film thickness for column was 0.25 *μ*m. Ultrahigh-purity helium was used as carrier gas (1 mL min^−1^). The temperature of the injector and detector was at 230°C. The oven temperature was set at 40°C, held for 5 min, and increased to 230°C at 5°C min^−1^ and was held at 230°C for a total run time of 60 min. Molecular weight, retention time, and percent likeness to the reference compounds from the National Institute of Standards and Technology (NIST) library were recorded to give tentative structures of volatile compounds.

#### 2.4.4. Characterization of Organic Acids Using HPLC-UV

The method described by Ergönül and Nergiz [20] was used. Fresh *V. infausta* fruit samples (20 g) were thawed at ambient temperatures. The seed was removed, and the pulp and peel were crushed using pestle and mortar. Water–methanol mixture (75:25 *v*/*v*) (10 mL) was added, and the slurry was centrifuged at 25°C at 3500 rpm/min for 30 min, using a Biobase BKC-MH12-B centrifuge (Biobase Meihua, Jinan, Shangdong, China). The upper phase was filtered through a Whatman no. 2 filter paper. Centrifugation was repeated three times and filtrates collected. The combined filtrates were again filtered through a Supelco Discovery DSC-18 filter. For separation and quantification of organic acids, a Thermoscientific UHPLC coupled with a UV absorbance detector set at 214 nm was used. Chromatographic separation was performed on an ion-exchange organic acid column (300 × 8 mm, i.d.). The mobile phase was 0.1% (*w*/*v*) phosphoric acid in distilled water (HPLC grade) with a flow rate of 0.8 mL/min. Aliquots (50 *μ*L) of the individual standard solutions (1000 pm) of ascorbic, acetic, formic, lactic acid, and propionic acid, previously prepared in the mobile phase consisting of 0.1% (*w*/*v*) phosphoric acid solution, were injected onto the column, and their retention times were determined. For obtaining the calibration curve, mixtures of the standards of selected concentration were injected into the HPLC, and their chromatograms were obtained. After injection of the fruit sample extracts, chromatographic peaks were identified by comparing the retention times of the samples with that of the standards. Concentrations of organic acids were estimated from the peak areas, based on the peak area of the standard.

### 2.5. Statistical Analysis

The results were expressed as mean ± standard deviation of replicate measurements, and one-way ANOVA to compare different treatments was done using the GraphPad Prism software version 7. Significant differences were determined at *p* ≤ 0.05.

## 3. Results and Discussion

### 3.1. Phytochemicals in *V. infausta*

Table 2 shows the types of phytochemicals present in two different extracts (water and methanol) of the *V. infausta* fruits. These were mainly phenols, tannins, flavonoids, coumarins, alkaloids, anthocyanins, and terpenoids. Alkaloids were detected in the methanol extract but not in the water extract. This can be explained by the differences in solvent properties and extraction efficiencies. The consumption of diets rich in flavonoids, phenolic acids, and lignans could reduce the risk of chronic diseases such as cardiovascular disease, cancers, osteoporosis, and neurodegenerative diseases such as Alzheimer's disease, which are prevalent among the older people [1]. Alkaloids have antioxidant, anxiolytic, anti-inflammatory, antidepressant, and anticholinesterase properties [21]. Presence of phytochemicals in wild fruits was also observed in previous investigations by Muchuweti et al. [22] who reported the presence of alkaloids in *Uapaca kirkiana* and Dabesor et al. [23] who reported the presence of glycosides, tannins, and alkaloids in *Cocos nucifera.*

The study also observed the presence of coumarins and terpenoids. Srikrishna et al. [24] reported that coumarins possess anti-inflammatory, antimicrobial, anticancer, anti-HIV, anti-TB, anticonvulsant, anticoagulant, and antioxidant properties. Previously, Gwatidzo et al. [3] conducted a study on in vitro anti-inflammatory activity of the *V. infausta* fruits and revealed that terpenoids, which are found in the unsaponifiable fraction, possessed anti-inflammatory activity. They inhibited the development of chronic joint swelling. The study also revealed the presence of tannins in *V. infausta* fruits. Tannins are a class of phytochemicals that have both negative and positive effects on health; for example, they have antinutritional properties, mutagenic, and hepatotoxic effects [25, 26]. They reduce the bioavailability of nutrients in the gut by forming complexes with calcium, magnesium, phosphorus, proteins, and carbohydrates rendering them unavailable for utilization by the body [25]. Bhebhe et al. [18], however, stated that at safe levels, tannins reduce high glucose levels in blood, scavenge-free radicals, and activate antioxidant enzymes in vivo.

**Figure 1:**
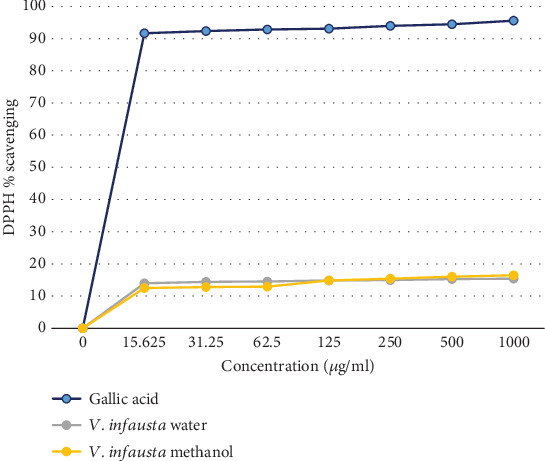
DPPH scavenging activity of *V. infausta* dried fruit pulp+peel.

The color intensity of phytochemicals in the methanolic extract was stronger than in the aqueous extracts. This is in agreement with Mwamatope et al. [27] who noted that generally methanolic extracts contained most of the compounds as compared to aqueous extracts. This was evidenced by the concentration of different phytochemicals in the *V. infausta* fruits (Table 3). There was a significant difference in the concentration of total phenolic compounds (*p* ≤ 0.05), total flavonoids (*p* ≤ 0.05), and total tannins (*p* ≤ 0.05) of methanolic versus aqueous extracts. The methanolic extracts had significantly higher concentrations of each phytochemical than the aqueous extract. This can be attributed to the polarity of methanol and the higher solubility of these compounds in methanol than in the aqueous (water) solvent [28, 29]. The TPC of *V. infausta* fruit pulp (128–170 mg GAE 100 g^−1^ DW) in this study was lower than what was reported by [27] (843–965 mg GAE 100 g^−1^) which was most probably due to the differences in the nature of samples (fresh versus dried), geographical location, and methods of analysis. In a study conducted by Laryea et al. [30], drying caused a reduction in phytochemicals in dandelion leaves. This is in agreement with other authors who propounded that drying can cause chemical changes in the structure of polyphenols or cause them to combine with other plant components such as proteins making their extraction difficult resulting in low recoveries [31]. From the study conducted by Laryea et al. [30], although fresh leaves had the highest TPC, freeze and solar drying retained most of the phenols as compared to oven drying. The TPC in the current study was higher than the one found by Mausse et al. [32] of 52.8 mg GAE 100 g^−1^ in the *V. infausta* fruit pulp. The antioxidant activity of the fruits as shown in Figure 1 was below 20%, and this could be attributed to the plant/tree parts, nature of the sample (fresh or dried), and type of extract used. The graph showed that the *V. infausta* fruits had an antioxidant activity. It is important to note that the scavenging activity graph for methanolic extract was almost the same as that of aqueous extract; however, the scavenging activity was below 20% for the fruits due to the form of the samples used; that is, dried samples and also the fruit have generally lower phytochemicals as compared to the roots, leaves, and bark which are commonly used in treatment of various ailments [1]. This is in agreement with a study done by Touré et al. [33] where there was comparison on the phytochemical and antioxidant activities of *Parkia biglobosa* leaves, fruit pulp, bark stem, and roots. The leaves had the highest polyphenol content (14.68), followed by the stem barks (11.69), roots (9.09), and lastly the fruit pulps (1.12 ± 0.43 mg GAE/g). Although the fruits have a low scavenging activity, they still possess health benefits to humans, as traditionally they are used to treat parasitic worms [1].

### 3.2. Characterization and Quantification of *V. infausta* Fruit Phytochemicals Using the HPLC Method

Phenolic acids detected in the *V. infausta* fruit pulps include caffeic acid, gallic acids, vanillin, *p*-coumaric acid, and quercetin (Table 4). The most abundant phytochemical in *V. infausta* fruits was gallic acid (10.23 ± 0.04 mg 100 g^−1^) followed by quercetin (5.87 ± 0.60 mg 100 g^−1^), vanillin (2.92 ± 0.06 mg 100 g^−1^), caffeic acid (0.23 ± 0.00 mg 100 g^−1^), and *p*-coumaric acid (0.10 ± 0.00 mg 100 g^−1^). Gallic acid is a natural phenolic compound found in fruits and medicinal plants which offers health benefits due to its antioxidant, anti-inflammatory, and antineoplastic properties. This compound is said to have therapeutic activities in neuropsychological, gastrointestinal, metabolic, and cardiovascular diseases [34]. Caffeic acid is also a phenolic acid which has antioxidant, anti-inflammatory, and anticarcinogenic activity [35]. Previous in vitro and in vivo studies have demonstrated the anticarcinogenic activity of this compound against an aggressive type of cancer called hepatocarcinoma (HCC). Vanillin and quercetin were the two flavonoid compounds detected in the *V. infausta* fruit pulp. Vanillin is a specialized metabolite that has both industrial applications and health benefits. It is used as a flavor food additive in the form of vanilla extract and has the following biological activities: anticancer, neuroprotective, antibiotic potentiation, and antiquorum sensing [36]. David et al. [37] reported that quercetin has antioxidant properties and has been shown to offer protection against osteoporosis, lung cancer, and cardiovascular diseases. In vivo and in vitro studies have shown that *p*-coumaric acid is a phenolic acid that has scavenging and antioxidative properties in the reduction of oxidative stress and inflammatory reactions. Due to the nutritional health benefits offered by *V. infausta* fruits, they can be used as a potential nutraceutical both in the food and pharmaceutical industries.

### 3.3. Characterization of Volatile Compounds in *V. infausta* Fruits Using the GC-MS

Table 5 shows the different types volatile organic compounds that were identified in the *V. infausta* fruit pulp after extraction with methanol, ethanol, and acetone. Methanolic extracts contained more volatile compounds compared to the other solvents, such as aldehydes, fatty acids, carboxylic esters, alcohols, steroids, and alkane hydrocarbons. Volatile compounds responsible for flavor and other food properties identified in this study include 5-hydroxymethylfurfurals, fatty acids such as ethyl-octadecanoic acid, methyl stearate, methyl- and ethyl-hexadecanoic acid, and methyl-2-furoate. Some compounds detected are similar to the ones identified by Raice et al. [5], which included hexanoic acid, octanoic acid, fatty acid methyl and ethyl esters, ethyl hexanoate, ethyl octanoate, methyl hexanoate, and methyl octanoate. Methyl stearate, hydroxymethyl furfurals, and methy-2-furoate were only identified in the current study. This could be due to differences in the soil type, climatic conditions, growth as well as biochemical factors like maturation stage, and method of harvesting the fruits [6, 38]. Generally, the identified volatile compounds are used as food additives, that is, food and beverage flavors, preservatives, anti-inflammatory agents, antimicrobial agents, emulsifiers, and stabilizers. This shows that *V. infausta* fruit pulps can be extracted and concentrated to make different food additives.

### 3.4. Characterization of Organic Acids Present in *V. infausta* Fruits

Table 6 shows that the fruit pulp of *V. infausta* contained formic acid (68.25 mg/kg) and ascorbic acid (297.3 mg/kg). Lactic, propionic, and acetic acids were not detected. Formic acid is known for its antimicrobial properties and is widely used as a preservative in the food industry [39]. Its mode of action involves infiltration of the bacterial cell at pH 7, dissociation to lower the cytoplasmic pH, and inactivating decarboxylases and catalase [40]. The FAO/WHO Expert Committee on Food Additives established an acceptable daily intake of formic acid of 0–3 mg/kg body weight [41]. This shows that *V. infausta* can be a potential source of formic acid which can be used as a natural food additive in beverages. The ascorbic acid content in the *V. infausta* fruit pulp was 297.3 mg/kg which is almost the same as the upper intake level for vitamin C of 2000 mg/day [42]. This shows that *V. infausta* fruits is a good source of ascorbic acid which is beneficial for the health of consumers. Ascorbic acid is an antioxidant that aids metabolism and enhances iron bioavailability by maintaining nonheme iron in the ferrous state [43]. Ascorbic acid is also thought to reduce the severity and duration of common cold symptoms by acting as an antihistamine and boosting immune response. In vitro, ascorbic acid destroys histamine by breaking the imizadole ring structure of the molecule, and in vivo, the plasma histamine concentrations are reduced by 40% in healthy adults within 2 weeks when 2 g/day ascorbic acid is taken in the form of vitamin C [44]. Schlueter and Johnston [43] further reported that ascorbic acid consumption was linked to a reduction in risk of cardiovascular diseases and some types of cancers. In this current study, citric, malic, and tartaric and succinic acids were not determined due to lack of standards. However, according to the literature, *V. infausta* fruits on average contain 6.2 g/kg citric acid, 2.1 g/kg malic acid, and 0.1 g/kg succinic acid [45]. This wild fruit therefore contains higher concentrations of citric acid compared to exotic counterparts such as oranges (4.5 g/kg) and pineapples (2.2 g/kg) but lower than grapes (13.1 g/kg) and lime (41.2 g/kg) [46].

Organic acids are involved in various metabolic pathways as intermediate or end products in humans; for example, citric, malic, succinic, fumaric, and oxalic acids play a key role in the Krebs cycle [47]. Organic acids also influence the organoleptic properties of plant foods, microbial stability, and product consistency [48]. They are used as food additives in the manufacture of beverages, including fruit and vegetable juices, for example, citric, malic, and tartaric acids (acidulants) and ascorbic acid (antioxidant) [49].

## 4. Conclusion

It can be concluded from the study that *V. infausta* fruits is rich in bioactive compounds such as phenolic compounds, flavonoids, and organic acids that have both pharmacological and food industrial applications. Some of these compounds are known to be beneficial to health indicating that extracts of the fruit can be used as ingredients for formulating some novel functional foods. The profile of volatile organic compounds can also potentially be used to enhance the flavor of foods and beverages. It is recommended that further work be done to investigate the commercial application of the fruit.

## Figures and Tables

**Table 1:** Parameters for determination of phytochemicals using HPLC.

	**Wavelength (nm)**	**Temperature (°C)**	**Injection volume (*μ*L)**	**Solvent A**	**Solvent B**	**Elution**	**Flow rate (mL/min)**
Gallic acid	270	35	1	Water/phosphoric acid (98:2 *v*/*v*)	Acetonitrile	Isocratic (60% A+40% B)	0.5
Caffeic acid and vanillin	300	35	5	Water/phosphoric acid (98:2 *v*/*v*)	Methanol	Gradient: 0 min, 10% B; 2 min, 10% B; 22 min, 90% B; 27 min, 90% B; 28 min, 10% B	0.5
Coumaric acid	285	35	5	Water/phosphoric acid (98:2 *v*/*v*)	Methanol	Gradient: 0 min, 10% B; 2 min, 10% B; 22 min, 90% B; 27 min, 90% B; 28 min, 10% B	0.5

*Note:* For caffeic acid, gallic acid, vanillin, and coumaric acid extraction: *V. infausta*: 10.0 g; refluxed with methanol/water (50:50 *v*/*v*) for 16 h using Soxhlet; evaporated to dryness under vacuum in rotary evaporator; dissolve in 10 mL methanol, filter, and inject into HPLC.

**Table 2:** Qualitative detection of phytochemicals in *V. infausta* fruit pulp+peel.

**Solvent**	**Phenols**	**Tannins**	**Flavonoids**	**Quinones**	**Anthraquinones**	**Coumarins**	**Alkaloids**	**Terpenoids**	**Glycosides**	**Anthocyanins**
Water	+	+	++	−	−	+++	−	+	−	+
Methanol	++	+	+	−	−	++	+++	+++	−	+

*Note:* + present in low amounts, ++ present in moderately high amounts, +++ present in very high amounts, − absent.

**Table 3:** Content of selected phytochemicals in *V. infausta* fruit pulp+peel in water and methanol extracts.

**Phytochemical**	***V. Infausta* water extract (** **n** = 3**)**	***V. Infausta* methanol extract (** **n** = 3**)**
Total phenols mg/100 g	128 ± 0.33^a^	170 ± 0.29^b^
Flavonoids mg/100 g	18 ± 0.03^a^	46 ± 0.02^b^
Tannins mg/100 g	3 ± 0.00^a^	7 ± 0.00^b^

*Note:* Means ± SD that do not share a letter in rows are significantly different *p* ≤ 0.05.

**Table 4:** Phytochemical content in *V. infausta* fruit pulp+peel.

**Fruit**	**Gallic acid (mg/100 g)**	**Caffeic acid (mg/100 g)**	**Vanillin (mg/100 g)**	***p*-Coumaric acid (mg/100 g)**	**Quercetin (mg/100 g)**
*V. infausta*	10.23 ± 0.04	0.23 ± 0.00	2.92 ± 0.06	0.10 ± 0.00	5.87 ± 0.60

*Note:* Results show mean ± standard deviation (SD) of duplicates.

**Table 5:** Tentative structures of volatile compounds detected in *V. infausta* fruit pulp after extraction with methanol, ethanol, and acetone.

**Compound detected**	**Compound type**	**Solvent used for extraction**	**Retention time**	**Properties/uses of the compound**
Furfural	Aldehyde	Methanol	5.80	Flavoring, inks, plastic manufacture, antacids, adhesives, fungicides, and fertilizers
2-Furanmethanol	Alcohol		6.43	Flavoring, sealants, and cement manufacture
2,5-Furandione, dihydro-3-methylene	—		8.61	—
2,5-Furan dicarboxyaldehyde	Aldehyde		12.43	Flavoring/odor agent
Methyl 2-furoate	Carboxylic ester		12.89	Flavoring agent in nuts and coffee etc., perfuming agent in cosmetics
4-H-Pyran-4-one,2.3-dihydro-3.5-dihydroxy-6-methyl	—		14.66	Mutagen antimicrobial, antioxidant, and anti-inflammatory
4-H-Pyran-4-one,3,5-dihydroxy-2 methyl	—		15.78	—
5-Hydroxymethyl furfural	—		17.36	Food additives, antimicrobial, preservative, flavoring agents
*n*-Hexadecanoic acid	Fatty acid		33.81	Flavoring agent, cosmetics, enzyme inhibitors, agricultural chemicals, lubricants
Stigmastan-3,5-diene	Steroid		54.44	Antimicrobial and antioxidant
		Ethanol		
Undecane	Alkane hydrocarbon		12.7	Cosmetics, antioxidant, antiallergy
4-H-pyran-4-one,2,3-dihydro-3,5-dihydroxy-6-methyl	—		14.4	Mutagen antimicrobial, antioxidant, and anti-inflammatory
Dodecane	Alkane hydrocarbon		15.8	Antioxidant in cosmetic products
5-Hdroxymethyl furfural			16.94	Food additives, antimicrobial, preservative, flavoring agents
Tridecane	Alkane hydrocarbon		18.64	Manufacture of paraffin products, rubber, and paper processing
Napthalene,1,2,3,4-tetrahydro-1,4-dimethyl	Tetralin		19.25	Solvent, paint, varnish, and rubber industries
Tetradecane	Alkane hydrocarbon		21.29	Skincare products and organic solvent
Pentadecane	Alkane hydrocarbon		23.79	Neutral, potential biomarker in foods
Hexadecane	Alkane hydrocarbon		25.16	Preparation of emulsions, volatile oil component
Heptadecane	Alkane hydrocarbon		28.41	Essential oil
Octadecane	Alkane hydrocarbon		30.54	Cosmetics
Nonadecane	Alkane hydrocarbon		32.58	Flavor agent in cosmetics and perfumes
Hexadecanoic acid, methyl ester	Fatty acid		33.12	Perfumes and cosmetics
*n*-Hexadecanoic acid	Fatty acid		33.78	Perfumes, cosmetics, flavoring agent, food additive adhesives, sealants, agricultural chemicals
Eicosane	Aliphatic hydrocarbon		34.53	Candle and paraffin waxes, cosmetics, lubricants, petrochemical industry
10,13-Octadecanoic acid, methyl ester	Fatty acid		36.45	Skin care, anti-inflammatory, antimicrobial properties, soap manufacture
9-Octadecanoic acid, methyl ester	Fatty acid		36.46	Anti-inflammatory agent
Methyl stearate	Fatty acid methyl ester		36.90	Emulsifier and stabilizer
		Acetone		
4-H-Pyran-4-one,2,3-dihydro-3,5-dihydroxy-6-methyl	—		14.42	Mutagen antimicrobial, antioxidant, and anti-inflammatory
5-Hydroxymethyl furfural	Cyclic aldehyde		16.91	Food additives, antimicrobial, preservative, flavoring agents

**Table 6:** Organic acid concentration in *V. infausta* fruit pulp.

**Organic acid**	**Concentration (mg/kg)**
Formic acid	68.25
Lactic acid	nd
Ascorbic acid	297.3
Propionic acid	nd
Acetic acid	nd

Abbreviation: nd, not detected.

## Data Availability

Data sharing is applicable to this article upon request to authors.
